# Optically Tunable Many‐Body Exciton‐Phonon Quantum Interference

**DOI:** 10.1002/advs.202404741

**Published:** 2024-08-29

**Authors:** Si‐Jie Chang, Po‐Chun Huang, Jia‐Sian Su, Yu‐Wei Hsieh, Carlos Jose Quiroz Reyes, Ting‐Hsuan Fan, Han‐Sheng Sun, Ai‐Phuong Nguyem, Te‐I Liu, Ho‐Wen Cheng, Ching‐Wei Lin, Michitoshi Hayashi, Chaw‐Keong Yong

**Affiliations:** ^1^ Department of Physics National Taiwan University Taipei 10617 Taiwan; ^2^ Institute of Atomic and Molecular Sciences Academia Sinica Taipei 106319 Taiwan; ^3^ International Ph.D. Program in Biomedical Engineering College of Biomedical Engineering Taipei Medical University New Taipei City 235603 Taiwan; ^4^ Department of Chemistry National Tsing Hua University Hsinchu 300044 Taiwan; ^5^ International Graduate Program of Molecular Science and Technology National Taiwan University Taipei City 106319 Taiwan; ^6^ Center for Condensed Matter Sciences National Taiwan University Taipei 10617 Taiwan; ^7^ Center of Atomic Initiative for New Materials National Taiwan University Taipei 10617 Taiwan; ^8^ National Center for Theoretical Sciences Taipei 10617 Taiwan

**Keywords:** fano resonance, floquet state, low‐dimensional semiconductors, optical manipulation, quantum interference

## Abstract

This study introduces a novel paradigm for achieving widely tunable many‐body Fano quantum interference in low‐dimensional semiconducting nanostructures, beyond the conventional requirement of closely matched energy levels between discrete and continuum states observed in atomic Fano systems. Leveraging Floquet engineering, the remarkable tunability of Fano lineshapes is demonstrated, even when the original discrete and continuum states are separated by over 1 eV. Specifically, by controlling the quantum pathways of discrete phonon Raman scattering using femtosecond laser pulses, the Raman intermediate states across the excitonic Floquet band are tuned. This manipulation yields continuous transitions of Fano lineshapes from antiresonance to dispersive and to symmetric Lorentzian profiles, accompanied by significant variations in Fano parameter *q* and Raman intensity spanning 2 orders of magnitude. A subtle shift in the excitonic Floquet resonance is further shown, achieved by controlling the intensity of the femtosecond laser, which profoundly modifies quantum interference strength from destructive to constructive interference. The study reveals the crucial roles of Floquet engineering in coherent light‐matter interactions and opens up new avenues for coherent control of Fano quantum interference over a broad energy spectrum in low‐dimensional semiconducting nanostructures.

## Graphical abstract

Leveraging Floquet engineering, the remarkable tunability of Fano resonance in 1D nanostructures is demonstrated, even when the original discrete and continuum states are separated by over 1 eV, leading to the continuous transitions of Fano lineshapes from antiresonance to dispersive and to symmetric Lorentzian profiles, accompanied by significant variations in Fano parameter *q* and Raman intensity spanning 2 orders of magnitude.

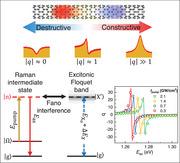

## 1 Introduction

**Figure 1:**
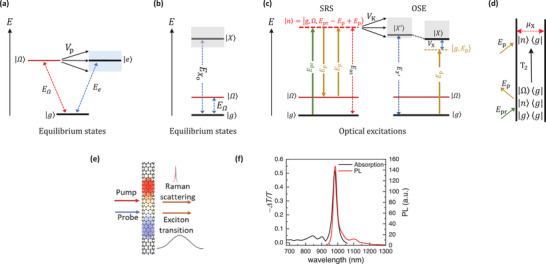
Schematics of Fano quantum interference. a) Classical model of Fano interference between coupled discrete phonon |Ω〉 and continuum state |*e*〉 with closely matched excitation energies. The blue‐shaded area shows the bandwidth of the continuum. b) Excitation energy of bare exciton |*X*〉 and discrete phonon |Ω〉 states in low‐dimensional semiconductors. The grey‐shaded area shows the bandwidth of the exciton continuum. c) Schematic diagram describing the optically‐driven quantum interference arising from the phonon Raman intermediate state |*n*〉 coupled to the excitonic Floquet band |*X*′〉 through electron‐phonon coupling *V*_K_ (black double arrow). Under optical excitation, the Floquet states |*g*, *E*_p_〉 hybridize with |*X*〉 through optical Stark effects (OSE) governed by electron‐photon coupling *V*_X_ (indicated by blue double arrow), leading to energy blueshift of the excitonic Floquet states |*X*′〉 (right panel). The interplays between electrons, phonons, and photons lead to rich stimulated Raman scattering (SRS) phenomena (left panel), a four‐wave mixing process. Following the generation of vibrational coherence by *E*_p_ and *E*_pr_, interactions with another *E*_p_ give rise to third‐order phonon Raman intermediate state |*n*〉, an admixture of electronic and phonon state |*g*, Ω, *E*_pr_ − *E*_p_ + *E*_p_〉, with energy depends on *E*_pr_ and *E*_p_ in the 3 field‐matter interactions. By employing a broadband probe with energy spans across exciton resonance, and controlling the pump energy, the outgoing Raman scattered photon, *E*_as_, can be tuned to align with the exciton resonance. d) The double‐sided Feynman diagram showing the evolution of the density matrix during the SRS described in (c). Arrows pointing into (away from) the diagram denote the absorption (emission) of photons. The horizontal dashed red arrow shows the overlap of bra and ket states mediated by the transition dipole moment μ_X_ to give the third‐order polarization. *T*_2_ describes the dephasing rate of vibrational coherence |Ω〉〈*g*| e) Illustration of the ultrafast pump‐probe spectroscopy on SWCNTs. f) The absorption (− Δ*T*/*T*, black solid line) and emission (PL, red solid line) spectra of semiconducting SWCNTs.

Fano interference controls the optical transition, scattering, and charge transport in solids.^[^ ^1^, ^2^, ^3^, ^4^ ^]^ This phenomenon has been ubiquitously observed across various domains in solid‐state systems featuring prominent many‐body interactions and closely matched excitation energies between discrete states and a continuum of states, including graphene,^[^ ^5^, ^6^ ^]^ metallic carbon nanotubes,^[^ ^7^, ^8^ ^]^ topological insulators,^[^ ^9^, ^10^ ^]^ plasmonic nanoparticles,^[^ ^11^, ^12^ ^]^ semiconductor nanostructures,^[^ ^13^, ^14^ ^]^ and strongly correlated electron systems.^[^ ^15^, ^16^ ^]^ Traditionally explored in the linear regime under low excitation intensities, as initially proposed by Fano,^[^ ^1^, ^2^, ^3^ ^]^ several recent investigations into nonlinear Fano quantum interference in low‐dimensional nanostructures have revealed intriguing ultrafast tunability of Fano resonance.^[^ ^17^, ^18^, ^19^, ^20^ ^]^ However, these studies remain confined within classic atomic Fano frameworks, focusing on tuning the optical transitions of the coupled quantum states with closely matched excitation energies, as illustrated in **Figure** **1**a. An exciting prospect emerges if Fano quantum interference can be optically driven and manipulated even when the discrete states are energetically remote from the continuum band (Figure 1b), going beyond the conventional Fano paradigm. This scenario would enable unambiguous tuning of Fano resonance across a broad energy range on an ultrafast timescale.

Low‐dimensional solid‐state systems provide a unique platform to test this concept. It is well‐established that the interplay of strong light‐matter couplings and many‐body electron‐phonon interactions in these systems can generate novel Floquet bands that are evenly spaced by the driving photon energy, and a remarkable array of exotic quantum phenomena.^[^ ^21^, ^22^, ^23^, ^24^ ^]^ Notably, by optically driving phonon and electron transitions using femtosecond optical pulses, band structure, and electronic correlation can be tailored, leading to the observation of light‐driven magnetism,^[^ ^25^, ^26^ ^]^ topological phase transition,^[^ ^24^, ^27^, ^28^ ^]^ charge density wave order,^[^ ^29^, ^30^ ^]^ and superconductivity.^[^ ^31^, ^32^ ^]^ Despite these advancements, the realization of an optically‐tunable many‐body exciton‐phonon Fano system, which enables control over the quantum interference between energetically remote discrete phonon modes and continuum electronic excitation, has remained elusive until now. Such dynamic quantum controls under femtosecond optical excitations not only give rise to rich many‐body Floquet states but also allow precise tuning of the phonon Raman intermediate states to closely align with the excitonic Floquet band, as illustrated in Figure 1c. Within the framework of strong electron‐phonon coupling, this scenario, in principle, can lead to rich quantum interference between optically driven phonon Raman intermediate states and excitonic Floquet band, even when the bare phonon states are energetically remote from the exciton states and qualitatively distinct from the classical atomic Fano system. However, experimental observation of light‐driven many‐body Fano resonance remains challenging, requiring spectroscopic techniques with the capacity to simultaneously control and probe the dynamics of both phonon and electronic degrees of freedom.

Here, we demonstrate that driving the phonon and excitonic excitations by femtosecond laser can profoundly modify the Fano quantum interference: it eliminates the conventional requirement of closely matched energy levels between the discrete and continuum band of states typically associated with the classical atomic Fano system, and lead to a rich set of quantum interference phenomena within the photon‐dressed quantum states. To reveal the optically controllable many‐body Fano effects, we study high‐purity *M*‐(6,5) semiconducting single‐walled carbon nanotubes (SWCNTs) using energy‐dependent pump‐probe spectroscopy, as shown in Figure 1e. We coherently drive the exciton transition and phonon Raman scattering using pump photons at an energy below the one‐exciton transition. By systematically tuning the quantum pathways of phonon Raman intermediate states across the resonance of the excitonic Floquet band, as illustrated in Figure 1c, we observe the Fano lineshapes evolve continuously from antiresonance to dispersive feature and to symmetric Lorentzian, signifying a shift of interference strength from destructive to constructive interference between the excitonic Floquet state resonance and the phonon Raman intermediate state transition, even when the bare phonon and exciton states are separated by at least 1 eV. We further show that a subtle shift of excitonic Floquet resonance, achieved by controlling the driving pump intensity, can dramatically modify the Fano lineshapes, and underscore the ultrasensitive nature of quantum interference on the competing interference pathways. Unlike the conventional Fano resonance, which arises from direct coupling between bare discrete states and continuum band, our results demonstrate the high tunability of Fano quantum interference between excitonic Floquet states and phonon Raman intermediate states, where the quantum pathways of these photon‐dressed states can be manipulated by adjusting the intensity and frequency of driving pump. These findings highlight the intricate interplay among photons, electrons and phonons in the low‐dimensional nanostructures can offer new avenues for optical manipulation of quantum interference with potential applications in novel quantum optoelectronics and nanodevices.

## 2 Results and Discussion

To demonstrate the quantum controls of Fano resonances, we use highly purified *M*‐(6,5) semiconducting SWCNTs suspended in 1% m v^−1^ sodium deoxycholate (SDC) aqueous solution as our model system (Section S1, Supporting Information). Strong quantum confinements in SWCNTs lead to prominent excitonic transition and electron‐phonon interactions,^[^ ^33^, ^34^ ^]^ making them suitable candidates to explore light‐driven many‐body exciton‐phonon Fano interference. Figure 1f shows the absorption and emission of the sample, where the optical transition is dominated by the excitonic resonance ($E_{X_{o}}$) at 1.263 eV. To reveal the optically controllable Fano quantum interference, we drive the Raman scattering and exciton transitions using a femtosecond pump at energy (*E*_p_) below the exciton transition, and probe the pump‐induced optical signals using probe pulses with energy (*E*_pr_) spanning across the exciton resonance in a pump‐probe spectroscopy (Figure 1e). Within the strong light‐matter coupling frameworks, the interaction can be understood using Floquet theory,^[^ ^35^ ^]^ where the negatively detuned driving pump photon ($\Delta E\;=E_{p}\;-E_{X_{o}}$) produces a series of Floquet states |*g*, *E*_p_〉 that are evenly spaced by *E*_p_,^[^ ^22^, ^35^, ^36^, ^37^, ^38^ ^]^ where |*g*〉 is the ground states. Quantum hybridization between these unperturbed Floquet states |*g*, *E*_p_〉 and the bare exciton state (|*X*〉) through optical Stark effects (OSE) gives rise to excitonic Floquet state (|*X*′〉), with transition energy blueshifted from the bare exciton state,^[^ ^35^, ^37^, ^38^ ^]^ as illustrated in the right panel of Figure 1c. This phenomenon can be captured by ultrafast pump‐probe spectroscopy, where the excitonic optical Stark shifts produce derivative‐like pump‐probe features near the exciton resonance.^[^ ^36^, ^39^, ^40^, ^41^, ^42^, ^43^ ^]^ Under large detuning limits, the Floquet theory predicts the optical Stark shift (Δ*E_X_*) increases linearly with the pump intensity (*I*_pump_) and inversely with detuning energy (Section S2 of the Supporting Information).

In addition to the optical Stark effects, the interactions between photons, electrons, and lattice vibrations can lead to rich stimulated Raman scattering (SRS) phenomena. This four‐wave mixing (FWM) process, where an electromagnetic field is emitted by nonlinear polarization induced by 3 field‐matter interactions,^[^ ^44^, ^45^, ^46^ ^]^ is illustrated in the left panel of Figure 1c, and further detailed in the corresponding double‐sided Feynman diagram in Figure 1d. Notably, when the energy of the probe exceeds the pump, the detection of pump‐probe signals along the probe channel renders the measurements sensitive to the anti‐Stoke Raman scattering.^[^ ^44^, ^45^, ^46^ ^]^ This third‐order nonlinear process initiates with the generation of vibrational coherence |Ω〉〈*g*|, when the energy difference between probe and pump matches a bare phonon energy ( *E*_Ω_ = *E*_pr_  − *E*_p_). This vibrational coherence then interacts with another pump photon *E*_p_, leading to the third‐order phonon Raman intermediate states |*n*〉. These intermediate states, represented as |*g*, Ω, *E*_pr_ − *E*_p_ + *E*_p_〉, are admixtures of electronic state and phonon state with energy depends on *E*_pr_ and *E*_p_ in the 3 field‐matter interactions. The third‐order polarization in the probe direction results from the overlap of the evolving ket |*n*〉 and the ground vibrational state bra 〈*g*|, mediated by transition dipole moment μ_X_, as illustrated in the double‐sided Feynman diagram in Figure 1d. For Raman signals detected along the probe direction, energy and momentum conservations dictate the outgoing photon energy, the Raman scattering resonance (*E*_as_), matches the annihilated probe photon *E*_pr_, and is linked to pump and phonon energy *E*_Ω_ via the relation *E*_as_ = *E*_p_  + *E*_Ω_, as illustrated in the left panel of Figure 1c.^[^ ^44^, ^45^, ^46^ ^]^ The Raman intermediate states |*n*〉 are known to play crucial roles as quantum pathways in inelastic light scattering, as exemplified in resonant Raman scattering and Raman interference.^[^ ^44^, ^45^, ^47^ ^]^

Experimentally, we drive the Raman scattering by employing broadband probe pulses with energy spans across the exciton resonance and negatively detuned pump pulses with narrow bandwidth. By controlling *E*_p_, the excitonic Floquet resonance *E*_*X*′_ shifts slightly by a few meV (Section S2, Supporting Information), while the Raman scattering resonance *E*_as_ varies linearly with pump photon energy. Consequently, by utilizing femtosecond pump pulses as a control knob, we can tune the quantum pathways of the phonon Raman intermediate state |*n*〉 to align with the resonance of the excitonic Floquet state |*X*′〉, as exemplified in Figure 1c, which lies at the core of optically tunable many‐body exciton‐phonon Fano resonance.

**Figure 2:**
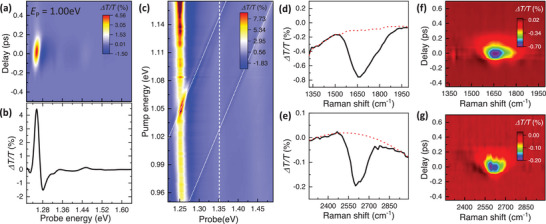
Energy‐dependent pump‐probe spectroscopy. a) Pump‐probe spectra of SWCNTs at room temperature. The color scale, vertical axis, and horizontal axis represent the transmission change Δ*T*/*T*, the pump‐probe time delay τ, and probe photon energy, respectively. The positive (negative) Δ*T*/*T* represents a decrease (increase) in absorption. The sample was excited with a linearly polarized pump at a photon energy *E*_p_ of 1.00 eV. b) At τ = 0 ps, the pump‐induced signals show spectral responses that are dominated by energy blueshift near the exciton resonance, signifying a blueshift of excitonic Floquet state |*X*′〉 under negatively detuned driving pump excitations. c) 2D plot of pump‐probe spectra at τ = 0 ps under different pump energies. The pump intensity was kept constant at 0.7 GW cm^−2^. The color scale, horizontal axis, and vertical axis represent transmission change Δ*T*/*T*, pump, and probe photon energy, respectively. Close to the exciton resonance, the optical responses are dominated by the optical Stark shift of exciton transition. Additionally, 2 prominent pump‐probe signals emerge diagonally across the 2D plot (indicated with white dotted lines), where the peak energy of the signals shifts by the same amount as *E*_p_, a defining signature of stimulated Raman scattering. d, e). Transient pump‐probe signals extracted along a line cut shown in (c) at a probe energy of 1.351 eV (vertical white dashed line). The horizontal axis corresponds to the Raman shift deduced from the energy difference between the probe and pump. The sharp features at energies close to 1650 cm^−1^ (d) and 2610 cm^−1^ (e) correspond to G‐mode and 2D‐mode phonon resonances in SWCNTs, respectively. f, g) Stimulated Raman scattering signals for G‐mode (f) and 2D‐mode (g) phonons at different τ (broad electronic transition backgrounds are subtracted) detected along *E*_pr_ = 1.351 eV probe channel.

**Figure** **2**a shows the pump‐probe signals generated by excitation with a linearly polarized pump at a photon energy of *E*_p_ = 1.00 eV, equivalent to a detuning energy of Δ*E*  = –0.263 eV. The photoinduced transmission changes are monitored with probe pulses with polarization parallel to the pump. Colors in Figure 2a represent the pump‐induced change of probe transmission Δ*T*/*T*, where the positive (negative) Δ*T*/*T* represents a decrease (increase) of pump‐induced absorption. The vertical and horizontal axes represent pump‐probe delay (τ) and probe energy, respectively. Strong Δ*T*/*T* occurs near τ  =  0 ps, diminishing significantly for τ exceeding 300 fs. This instantaneous response indicates that the photoinduced signals at τ = 0 ps are dominated by coherent optical Stark effects. The contribution from non‐coherent signals due to real carrier excitation, which typically lasts for many picoseconds,^[^ ^48^ ^]^ is negligibly small since we employed negatively detuned pump excitation. In Figure 2b, the pump‐probe spectrum at τ = 0 ps reveals the exciton absorption exhibiting a reduction below $E_{X_{o}}$ and an increase above $E_{X_{o}}$, corresponding to a blueshift of exciton resonance due to optical Stark effects. This suggests that the excitonic Floquet state |*X*′〉, under negatively detuned driving pump excitations, is blue‐shifted from the bare exciton transition |*X*〉.^[^ ^35^, ^36^, ^37^, ^38^, ^39^ ^]^ Figure 2c displays the 2D pump‐probe spectroscopy data at τ = 0 ps with the vertical and horizontal axes denoting the pump and probe energy, respectively. The color represents the photo‐induced change of the probe transmission Δ*T*/*T*. The pump intensity was kept constant at 0.7 GW cm^−2^. Close to the exciton resonance, the pump‐probe signals are primarily influenced by the optical Stark shift of exciton transition, with signals increasing as the detuning energy of the driving pump decreases, indicating that the blueshift of excitonic Floquet state |*X*′〉 becomes more pronounced with reduced detuning energy, consistent with the predictions based on Floquet theory (Figure S6, Section S2, Supporting Information).

In addition to the prominent signals originating from optical Stark effects near the exciton resonance, we observe 2 prominent pump‐induced signals emerging diagonally across the 2D plot in Figure 2c. Notably, the peak energies of the signals shift by precisely the same amount as the driving pump photon energy (white dotted lines), which are characteristic signatures of stimulated Raman scattering. This observation suggests that, apart from the excitonic optical Stark effects, the stimulated Raman scatterings also contribute to the pump‐probe signals. Quantitative analysis can be made by taking a line cut vertically along the probe axis in Figure 2c. For Raman scattering detected at a specific probe energy, energy and momentum conservations dictate the Raman scattering resonance emerges when the sum of the driving pump and phonon energy matches the probe photon (left panel of Figure 1c). Panel d and e in Figure 2 display the photo‐induced responses at a probe energy of *E*_pr_ = 1.351 eV (vertical white dashed line in Figure 2c), where 2 distinct pump‐probe signals are observed when the pump is tuned close to 1.15 eV and 1.03 eV. The horizontal axis corresponds to the Raman shift deduced from the energy difference between the probe and pump. Indeed, within these pump‐probe signals, we identify a broad background with sharp optical responses occurring at energies that coincide with the G‐mode (≈1600 cm^−1^) and 2D‐mode (≈2600 cm^−1^) phonon Raman in SWCNTs, as observed in previous studies.^[^ ^7^, ^49^ ^]^ These observations confirm that, in addition to the excitonic transition, the diagonal signals in the 2D pump‐probe spectroscopy data stem from stimulated Raman scatterings, where the outgoing photon energy *E*_as_ arising from such third‐order nonlinear polarization varies linearly with the driving pump photon, and coincides with the probe photon, as shown in Figure 2c.

Interestingly, instead of a symmetric Lorentzian lineshape, as typically expected for ordinary stimulated Raman scattering process,^[^ ^45^ ^]^ the Raman signals exhibit a prominent asymmetric dip, and persist throughout the temporal duration of pump‐probe overlaps, as displayed in Figure 2f,g. We can rule out the asymmetric dips resulting merely from the interplays of multiple interfering signals in the third‐order nonlinear responses, as such a scenario would entail observable changes in lineshapes with varying time delays.^[^ ^45^, ^46^, ^50^ ^]^ Instead, this intriguing “anti‐resonance” signature, where the Raman scattered photon *E*_as_ aligns closely with the exciton resonance, is an indication of Fano quantum interference between the transitions originating from the third‐order phonon Raman intermediate state |*n*〉 and the excitonic Floquet band |*X*′〉, mediated by electron‐phonon coupling *V*_k_ in SWCNTs. Such prominent many‐body exciton‐phonon Fano effects, even when the bare phonon and exciton resonances are separated by more than 1 eV, clearly indicate that the conventional Fano picture in atomic system (Figure 1a) cannot fully account for the quantum interference observed in low‐dimensional semiconducting nanostructures, and imply that the coupling between the excitonic Floquet states and third‐order phonon Raman intermediate states is of critical importance (Figure 1c).

**Figure 3:**
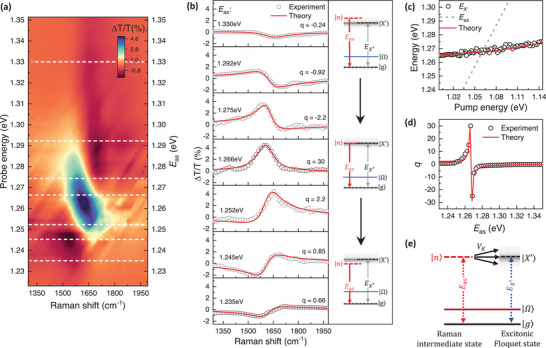
Raman spectra of the optically tunable many‐body Fano resonance. **a)** 2D plot of transient Raman spectra at various probe energies at τ = 0 ps. For SRS detected along the probe direction, the probe energy simply coincides with the Raman scattering resonance *E*_as_ (right vertical axis). The horizontal axis shows the Raman shift calculated from the energy difference between the pump and probe for each probe energy. The color scale represents transmission change Δ*T*/*T*. b) The transient Raman spectra extracted from the line cuts (white dashed lines) shown in (a) at various probe energies. Red curves are fitted with Fano lineshapes based on equation 1. The right panel shows the evolution of the Raman quantum pathways |*n*〉 and excitonic Floquet band |*X*′〉. c) Measured exciton resonance (black circles) as a function of driving pump energy for a driving intensity of 0.7 GW cm^−2^. The dependence can be nicely described by an inverse proportional relationship (red line). The dashed line shows the calculated Raman scattering frequency *E*_as_ as a function of pump energy. d) The Fano *q*‐factor extracted by fitting the Raman spectra based on Equation 1 (black circles) is in good agreement with the predictions based on Equation 2 (red solid line). e) Schematic diagram illustrating the effective coupling between the phonon Raman intermediate state |*n*〉 and excitonic Floquet states |*X*′〉 under driving pump irradiation. The interactions are governed by the electron‐phonon coupling constant *V_k_*.

To gain insight into the remarkably pronounced Fano resonance observed in SWCNTs, we focus on the G‐mode Raman by extracting the Raman spectra at different probe energies from the 2D pump‐probe spectroscopy data in Figure 2c. **Figure** **3**a shows the 2D plot of Raman spectra at various probe energies at τ = 0 ps. For SRS signals detected along the probe direction, the outgoing Raman scattering resonance *E*_as_ (indicated on the right vertical axis of Figure 3a) simply overlaps with the probe, following the principles of energy and momentum conservations in a FWM process (Figure 1c). On the plot, the horizontal axis represents the Raman shift derived from the energy difference between the pump and probe for each probe energy, while the color scale reflects the photoinduced change of probe transmission. We have subtracted the broad background using the third‐order polynomial fittings, in order to focus on the sharp Raman features (Section S3, Supporting Information). Intriguingly, we observe the Raman lineshapes vary rapidly as the outgoing Raman scattered photon *E*_as_ is scanned across the resonance of the excitonic Floquet state at *E*_*X*′_ = 1.267 eV. The Raman signals become diminishingly small when *E*_as_ is tuned further below *E*_*X*′_.

Figure 3b provides a closer examination of the Raman spectra at several representative *E*_as_, indicated by the dashed lines in Figure 3a. The lineshapes evolve from antiresonance to dispersive features, and to symmetric Lorentzian profiles, as *E*_as_ is tuned toward the exciton resonance. The evolution of these lineshapes reverses when *E*_as_ is tuned across the exciton resonance. Additionally, not only do the lineshapes transform with changing *E*_as_, but the Raman intensity also increases by at least one order of magnitude as *E*_as_ approaches *E*_*X*′_. Similar behaviours were observed in 2D modes Raman (Section S4, Supporting Information). Meanwhile, the exciton resonance merely shifts by 10 meV across the tuning window, as shown in Figure 3c. Besides these observations, a weak D‐mode Raman signal ≈1360 cm^−1^ becomes apparent when *E*_as_ is tuned close to *E*_*X*′_ (Section S3, Supporting Information). This signal is much weaker in intensity compared to the G‐mode Raman, indicating the high quality of our sample.

Since the Raman lineshapes vary rapidly as the Raman scattering pathways are tuned across the interband exciton transition by the driving pump, we consider the quantum interference arising from the hybridization between the discrete phonon Raman intermediate state |*n*〉 and excitonic Floquet band |*X*′〉, as illustrated in Figure 3e. The Raman spectra can be well‐described by the Fano lineshapes *I*(*E*):^[^ ^1^, ^2^, ^5^ ^]^

(1) $$I\;\left(E\right)=I_{o}\;\left(\frac{{\left[q\cdot \gamma_{\Omega^{\prime }}+\left(E-E_{\Omega^{\prime }}\right)\right]}^{2}}{{\left(E-E_{\Omega^{\prime }}\right)}^{2}+{\gamma_{\Omega^{\prime }}}^{2}}-1\right)$$

where *I*_o_, *q*, γ_Ω′_, and *E*_Ω′_ are fitting parameters related to the bare electronic scattering, Fano parameter, linewidth, and center frequency of the dressed phonon. Within the Equation 1 numerator, γ_Ω′_ and (*E* − *E*_Ω′_) describe the phonon and electronic weights in the hybrid wavefunction at different energies. The Fano parameter, *q*, characterizes the relative strength of dressed‐phonon Raman scattering and excitonic transitions.^[^ ^1^, ^2^ ^]^ Depending on the value of *q*, the lineshape can exhibit characteristics of Lorentzian (|*q*| ≫ 1, phonon Raman dominates), dispersion (|*q*| ≈ 1, comparable phonon and exciton contribution), or antiresonance (|*q*| ≪ 1, exciton excitation dominates). Figure 3b presents the calculated Fano lineshapes at various Raman scattering frequencies *E*_as_ through fitting the experimental data to Equation 1 (Section S5, Supporting Information). The critical parameter describing these lineshapes is the Fano parameter *q*, as displayed in Figure 3d. This parameter, derived from fitting the experimental data at different scattering frequencies using Equation 1, depends sensitively on the quantum pathways of the Raman intermediate state |*n*〉: it varies by at least 2 orders of magnitude as *E*_as_ is tuned closer to the resonance of excitonic Floquet state and changes signs as *E*_as_ crosses this resonance. In addition, the center frequency and linewidth of the dressed phonon also change with *E*_as_, signifying the hybrid phonon‐exciton nature of the Raman intermediate state is strongly renormalized by the tunable many‐body Fano quantum interference (Section S5, Supporting Information). These results demonstrated that the Fano interference between the transitions originating from the excitonic Floquet band |*X*′〉 and the third‐order phonon Raman intermediate state |*n*〉 can be continuously tuned from asymmetric antiresonance (|*q*| ≪ 1) to Lorentzian lineshapes (|*q*| ≫ 1), with the driving pump serving as a single control knob, even when the bare phonon state is energetically remote from the exciton band.

Quantitative analysis of the Fano parameter *q* provides more detailed information on the optically tunable many‐body Fano quantum interference. Previous studies have shown that the Fano parameter *q* is given by:^[^ ^1^, ^2^, ^5^ ^]^

(2) $$q=\frac{1}{\pi D\left(E\right)V_{k}}\times \frac{M_{\Omega }}{M_{X}}$$

where *V*_k_ is the exciton‐phonon coupling strength, and *D*(*E*) is the joint electron‐hole pair density of states at the energy corresponding to the Raman intermediate state (Section S6, Supporting Information). $M_{\Omega }$ and *M_X_* describe the matrix elements for phonon Raman scattering and interband electronic transition, respectively. For G‐mode phonon, the resonant Raman scattering process yields an energy‐dependent $M_{\Omega }$ that can be approximated in the form of $M_{\Omega }=\frac{B_{k}}{\left(E_{X^{\prime }}-E_{\mathrm{pr}}-i\gamma_{X}\right)\left(E_{X^{\prime }}-E_{p}-E_{\Omega }-i\gamma_{\Omega }\right)}$.^[^ ^51^, ^52^, ^53^ ^]^ Here *B_k_* is a fitting constant related to the joint electron‐ hole pair density of states, electron‐phonon couplings, and electron‐photon interactions. $\gamma_{\Omega }$ and γ_*X*_ denote the linewidth of bare phonon and exciton, respectively. For G‐mode Raman, $\gamma_{\Omega }$ typically assumes value on the order of ∼1 meV, which is notably smaller than $E_{\Omega }$.^[^ ^7^, ^54^ ^]^ The impacts of optical Stark effects on the exciton resonance are captured by *E*_X′_, which scales inversely with the detuning energy of the driving pump (solid line in Figure 3c). The interband exciton excitation, on the other hand, can be approximated in the form of $M_{X}=\frac{V_{X}}{E_{X^{\prime }}-E_{\mathrm{pr}}-i\gamma_{X}}$, where *V*_X_ is a fitting parameter related to the electron‐photon coupling.^[^ ^44^, ^55^ ^]^ In our case, we set γ_*X*_ to 45 meV, as extracted from the absorption spectrum (Figure 1f). It is noteworthy that *q* depends on the experimentally measured quantities *E*_p_, *E*_*X*′_ and $E_{\Omega }$, and is not sensitive to *D*(*E*), *V_k_*, *B*_k_ and *V*_X_ since the Raman activity $M_{\Omega }$ is proportional to the *D*(*E*), electron‐phonon, and electron‐photon couplings, cancelling the one in the denominator of Equation 2 (Section S6, Supporting Information).

Experimentally, we drive the system using a negatively detuned driving pump at energies below the exciton resonance, while the outgoing Raman scattered photon *E*_as_ from |*n*〉 is tuned through the exciton resonance *E*_*X*′_. For small $\gamma_{\Omega }$, $M_{\Omega }$ evolves rapidly when *E*_as_ is tuned close to *E*_*X*′_, changing signs as *E*_as_ is swept across *E*_*X*′_. On the other hand, *M_X_* shows a more gradual variation across the tuning window. Such distinct behaviors between $M_{\Omega }$ and *M_X_*, even when the incoming pump photon remains negatively detuned relative to the exciton resonance, lies at the center of the experimentally observed evolution of many‐body exciton‐phonon Fano interference: when the outgoing Raman scattered photon *E*_as_ is on resonance with excitonic Floquet resonance *E*_*X*′_, |*q*| ≫ 1, leading to symmetric Lorentzian lineshapes. Away from *E*_*X*′_, *q* varies rapidly, and changes sign as *E*_as_ is tuned across *E*_*X*′_, giving rise to asymmetric Fano lineshapes.

Direct comparison between the theoretical model and experiment can be achieved by extracting the real component from the solution to Equation 2. Figure 3d presents the fit of the Fano factor *q* based on Equation 2 that qualitatively reproduce the experimentally obtained values, considering the simplicity of our model. The excellent agreement between the fitted results from Equation 2 and experimental data further substantiates that the many‐body exciton‐phonon Fano resonance depends critically on the quantum mechanical amplitude, that is, magnitude and energy, of the Raman scattering and exciton transition, and can be controlled using a negatively detuned driving pump at energy below exciton resonance.

**Figure 4:**
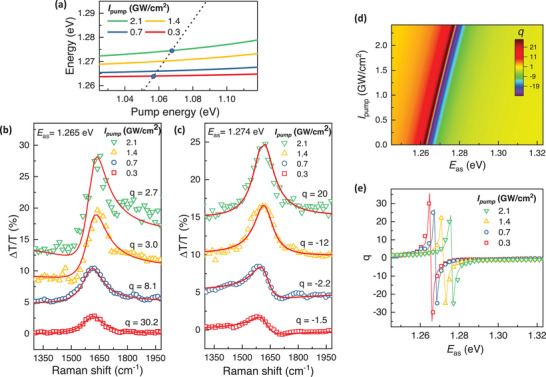
Pump intensity dependence of Fano resonance. a) Calculated exciton resonance as a function of driving pump energy at different driving intensities *I*_pump_ (solid lines). Depending on the pump intensity, the Raman scattering frequency *E*_as_ (black dashed line) intercepts with the exciton resonance at different energies. The blue circles show the resonant Raman scattering conditions for driving pump intensities set to 0.3 and 2.1 GW cm^−2^. b, c) Driving pump intensity‐dependent Fano lineshapes at (b) *E*_as_ = 1.265 eV and (c) *E*_as_ = 1.274 eV. By controlling the driving pump intensity, the Fano lineshapes for a given *E*_as_ varies from asymmetric dispersive feature to symmetric Lorentzian, corresponding to a change of interference parameter from |*q*| ≈ 1 to |*q*| ≫ 1. d) The Fano *q*‐parameter calculated based on Equation 2 for different driving pump intensities *I*_pump_. e) The Fano *q*‐parameter as a function of *E*_as_ at different excitation intensities. Symbols are *q* values obtained from fitting the Raman spectra based on Fano lineshapes described in Equation 1 and solid lines are fitting based on Equation 2.

Instead of modifying the detuning energy, the emerging many‐body exciton‐phonon Fano interference can also be manipulated by controlling the driving pump intensity (*I*_pump_) at energies below the exciton transition. Under negatively detuned driving pump excitation, the optical Stark shifts increase with higher excitation intensity, subsequently modifying the resonant Raman scattering conditions, as illustrated in **Figure** **4**a. As a result, the Fano lineshapes for Raman scattering resonance *E*_as_ tuned close to excitonic Floquet resonance *E*_*X*′_ can become highly sensitive to the driving pump intensity, as implied by Equation 2 (Figure 4d). To reveal the high sensitivity of Fano effects to exciton resonance, we compare Fano lineshapes at Raman scattering frequency *E*_as_ = 1.265 and 1.274 eV at different excitation intensities in Figure 4b,c, respectively. These energies match with the resonant Raman scattering conditions under excitation intensities of 0.3 and 2.1 GW cm^−2^ (blue circles in Figure 4a), as predicted by our theoretical model (Section S2, Supporting Information). Indeed, when *E*_X′_ aligns with *E*_as_, the Fano lineshapes are characterized by symmetric Lorentzian with |*q*| ≫ 1. When *E*_*X*′_ is slightly detuned from *E*_as_, the Raman spectra show asymmetric dispersive behaviors with |*q*| ≈ 1. Figure 4e shows the interference parameter *q* obtained by fitting the Raman lineshapes using Equation 1 (Section S4, Supporting Information), a result that quantitatively aligns with a fit of the theoretical model based on Equation 2. These results demonstrate the ultrasensitive nature of Fano interference can be fine‐tuned by simply controlling the driving pump intensity at an energy below exciton resonance, enabling ultrafast switching between asymmetric Fano lineshapes and symmetric Lorentzian profiles on the femtosecond timescale.

## 3 Conclusion

In summary, our study reveals that even when the original phonon |Ω〉 and exciton states |*X*〉 are separated by more than 1 eV scale, Floquet engineering of quantum states through femtosecond optical excitation aligns the phonon Raman intermediate states |*n*〉 with the excitonic Floquet band |*X*′〉, offering a unique platform to tune the Fano quantum interference that goes beyond the classical atomic Fano framework. The Fano lineshapes evolve dynamically from antiresonance to dispersive features and ultimately to Lorentzian profile, as the Raman quantum pathways are driven closer to the excitonic Floquet band, signifying a change of quantum interference strength from destructive to constructive interference. Our findings open up exciting opportunities to tune quantum interference between competing pathways across a broad energy spectrum, enabling controls of various nonlinear optical processes and electron dynamics in low‐dimensional systems on ultrafast timescales. Such quantum controls will make inelastic light scattering a more powerful tool for probing many‐body physics at nanoscales and enabling optimization of inelastic light scattering in quantum nanostructures for biological sensors and quantum optoelectronic applications. Future theoretical advancements will be essential to fully understand the microscopic mechanisms and dynamics of many‐body interactions in low‐dimensional nanostructures that drive the quantum interference phenomena at ultrafast timescales.

## 4 Experimental Section

### *M*‐(6,5) Semiconducting SWCNTs Preparation

The *M*‐(6,5), or left‐handed (6,5), or (+) (6,5), preparation includes 3 major steps: CoMoCAT suspension, (6,5) chirality extraction, and M‐(6,5) enantiomer extraction. The details of the separations were described as follows.

### CoMoCAT Suspension

10 mg of CoMoCAT SG65i powder (Chasm Signis Lot. MKCK1004) was tip sonicated in 50 mL of 1% m v^−1^ sodium deoxycholate (SDC; Sigma BioXtra) aqueous solution for 72 h. Subsequently, the dispersion was centrifuged at 140 000 g for 1.5 h, followed by the collection of the supernatant.

### (6,5) Chirality Extraction

A concentration step was started using 5% m m^−1^ dextran (DX; MW 70 kDa, TCI) and 10% m m^−1^ polyethylene glycol (PEG; MW 6 kDa, Alfa Aesar) to adjust SDC into 0.5% m v^−1^. The concentrated bottom phase was collected and mixed with PEG to introduce 0.5% m v^−1^ sodium dodecyl sulfate (SDS; Sigma‐Aldrich BioXtra) and adjust SDC to 0.05% m v^−1^. Multiple additions of 0.125 M HCl reduce the pH and induce the migration of large‐diameter SWCNTs to the PEG‐rich phase. The bottom was carefully collected, leaving the interface to avoid collecting impurities. One‐part mimic phase (0.05% m v^−1^ SDC, 0.5% m v^−1^ SDS, 15% m w^−1^ PEG) and one‐part bottom phase from the previous step were mixed with additional HCl to induce the migration of (6,5) species into the top phase, leaving the smaller diameter SWCNTs in the bottom phase. A single species was collected from the top phase, filtered through a 100 kDa MWCO tangential flow filtration (TFF) hollow fiber filter module, and concentrated using 1% m v^−1^ sodium cholate (SC) for enantiomeric separation. The absorption spectra were measured in every step to confirm the purity of large and small‐diameter species.

### M‐(6,5) Enantiomer Extraction

First, a concentration step was performed using 10% m m^−1^ PEG and 5% m m^−1^ DX to reduce (6,5) sample concentration in half. After a complete phase separation, the top phase without SWCNTs was discarded. Then the concentrated bottom phase was mixed with 7% m m^−1^ PEG and 1.5% m m^−1^ DX to achieve the optimal surfactant concentration (0.95% SC, 0.7% SDS, and 0.025% SDC). This condition will keep both enantiomers in the DX‐rich bottom phase. Then, small additions of SDS 10% were used to push *M*‐(6,5) to the PEG‐rich phase. The *M*‐(6,5) collected from the top phase was filtered through a 100 kDa MWCO TFF hollow fiber filter and concentrated in 1% m v^−1^ SDC. The absorption, fluorescence, and CD spectra were measured to confirm the concentration and purity of the samples.

### Ultrafast Pump‐Probe Spectroscopy

The pump‐probe spectroscopy study was based on a regenerative amplifier seed by a mode‐locked oscillator (Light Conversion PHAROS). The regenerative amplifier delivers femtosecond pulses at a repetition rate of 175 kHz and a pulse duration of ≈150 fs, which were split into 2 beams. One beam was used to pump an optical parametric amplifier and the other beam was focused onto a sapphire crystal to generate supercontinuum light (700–1050 nm) for probe pulses. The cross‐correlation of the pump and probe pulses has a full‐width half‐maximum close to ≈220 fs. The pump‐probe time delay was controlled by a motorized delay stage. The probe light was detected by a high‐sensitivity CCD line camera operated at 75 Hz. The pump and probe pulses were linearly polarized using a broadband Glan‐Thomson polarizer. The polarization of pump and probe pulses were parallel to each other in the measurements. The intensities ratio of the probe (*I*_probe_) and pump (*I*_pump_), *I*_probe_: *I*_pump_, is kept lower than 0.1 for all measurements. The experiment followed a transmission configuration with pump and probe pulses focused onto the sample separately using achromatic lens. The sample was loaded into a cuvette with an optical path length of 1 mm. All optical measurements were performed at room temperature.

## Conflict of Interest

The authors declare no conflict of interest.

## Author Contributions

S.‐J.C. and P.‐C.H. contributed equally to this work. The study was conceived by C.‐K.Y. C.‐K.Y. S.‐J.C. and P.‐C.H designed the experiments and carried out optical measurements and assisted by J.‐S.S., Y.‐W.H and T.‐H.F. C.‐K.Y., P.‐C.H, and S.‐J.C. analyzed the data and performed theoretical analysis assisted by Y.‐W.H. and M. H. C.J.Q. and C.‐W.L. prepared the high‐purity carbon nanotubes samples. H.‐S.S. prepared samples for fluorescence imaging, SEM imaging, AFM imaging, and Raman spectroscopy. A.‐P.N. carried out fluorescence imaging. H.‐W.C. conducted SEM imaging. T.‐I.L. conducted Raman measurements. C.‐W.L. performed AFM imaging and supervised fluorescence imaging, SEM imaging, and Raman spectroscopy measurements. C.‐K.Y. wrote the manuscript with input from all authors.

## Supporting information

Supporting Information

## Data Availability

The data that support the findings of this study are available from the corresponding author upon reasonable request.
