# Supplementary material for: Optically Tunable Many‐Body Exciton‐Phonon Quantum Interference
Source: Adv Sci (Weinh). 2024 Aug 29;11(40):2404741. doi: 10.1002/advs.202404741 (PMC11516097; doi:10.1002/advs.202404741)
Supplement: Supplementary file 1 — Supporting Information [file ADVS-11-2404741-s001.pdf]

ADVANCED SCIENCE
Open Access

Supporting Information

for *Adv. Sci.*, DOI 10.1002/advs.202404741

Optically Tunable Many-Body Exciton-Phonon Quantum Interference

*Si-Jie Chang, Po-Chun Huang, Jia-Sian Su, Yu-Wei Hsieh, Carlos Jose Quiroz Reyes, Ting-Hsuan Fan, Han-Sheng Sun, Ai-Phuong Nguyem, Te-I Liu, Ho-Wen Cheng, Ching-Wei Lin, Michitoshi Hayashi and Chaw-Keong Yong**

Supporting Information

Optically tunable many-body exciton-phonon quantum interference

Si-Jie Chang[†,1], *Po-Chun Huang*[†,1], *Jia-Sian Su*[1], *Yu-Wei Hsieh*[1], *Carlos Jose Quiroz Reyes*[2,3], *Ting-Hsuan Fan*[1], *Han-Sheng Sun*[2], *Ai-Phuong Nguyem*[2,4], *Te-I Liu*[2], *Ho-Wen Cheng*[2,5], *Ching-Wei Lin*[2], *Michitoshi Hayashi*[6,7,8], *Chaw-Keong Yong*[*,1]

[1]Department of Physics, National Taiwan University, Taipei 10617, Taiwan

[2]Institute of Atomic and Molecular Sciences, Academia Sinica, Taipei 106319, Taiwan

[3]International Ph.D. Program in Biomedical Engineering, College of Biomedical Engineering, Taipei Medical University, New Taipei City 235603, Taiwan

[4]Department of Chemistry, National Tsing Hua University, Hsinchu 300044, Taiwan

[5]International Graduate Program of Molecular Science and Technology, National Taiwan University, Taipei City 106319, Taiwan

[6]Center for Condensed Matter Sciences, National Taiwan University, Taipei 10617, Taiwan

[7]Center of Atomic Initiative for New Materials, National Taiwan University, Taipei 10617, Taiwan

[8]National Center for Theoretical Sciences, Taipei 10617, Taiwan

Section 1. Characterization of semiconducting SWCNTs

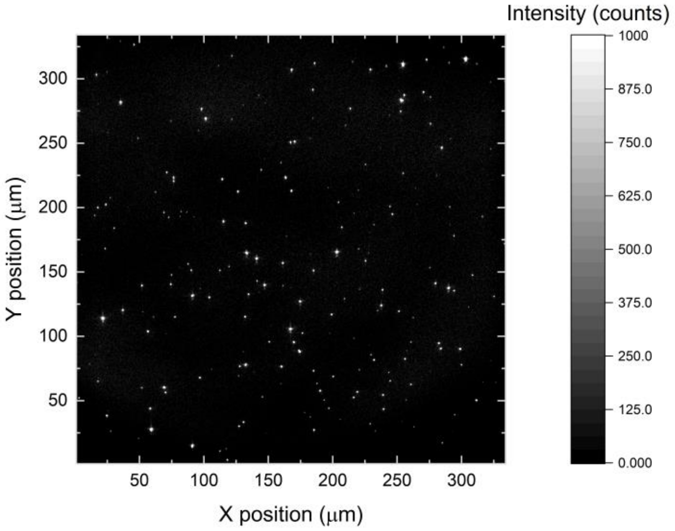

Figure S1. Short-wave infrared fluorescence microscopy of individualized SWCNT. The sample was dissolved in a 1% sodium deoxycholate aqueous solution. Several microliters of the solution were taken and drop-cast onto a coverslip, which was then attached to a microscope slide for imaging. The image was captured using a 561 nm excitation laser (100 mW excitation power; Cobolt, HÜBNER Photonics) and a deeply cooled silicon camera (50 s acquisition time; −80 °C; iKon-M 934) with a 950 nm filter cube. A 40× objective (CFI Plan Apo λD 40×/0.95, Nikon) was used to image the SWCNT fluorescence. The different brightness represents their different lengths of SWCNT.

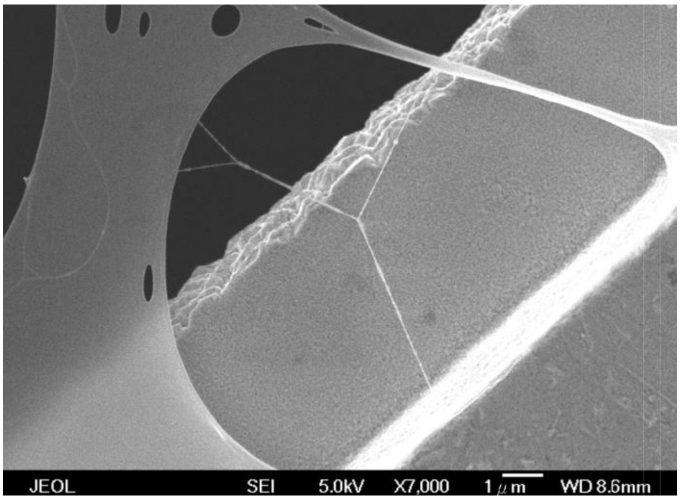

Figure S2. Scanning electron microscopy (SEM) image of SWCNTs. SEM image captured by JEOL JSM-6700F that was operated at an accelerating voltage of 5 kV. The individual SWCNTs have lengths ~100-200 nm. Bundled aggregation was formed on lacey TEM grid (LC305-CU, EMS) to produce ultralong SWCNT fiber after dried out. Individual SWCNTs are not possible to be observed using SEM because of its lower resolution.

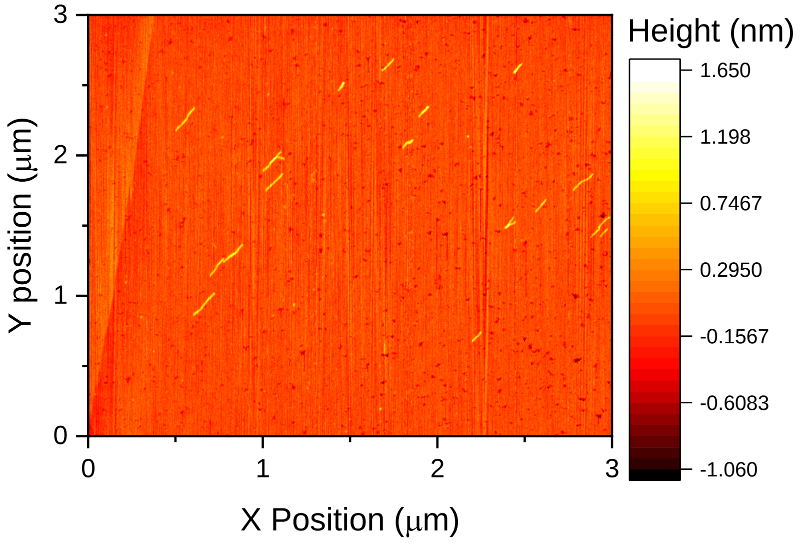

Figure S3. The atomic force microscopy (AFM) of of SWCNTs. The sample was deposited on Mica surface. The image was acquired using Veeco Icon model. The model of the AFM probe was TESPA. The scan rate was set to 1 Hz, and the pixel number was 1024.

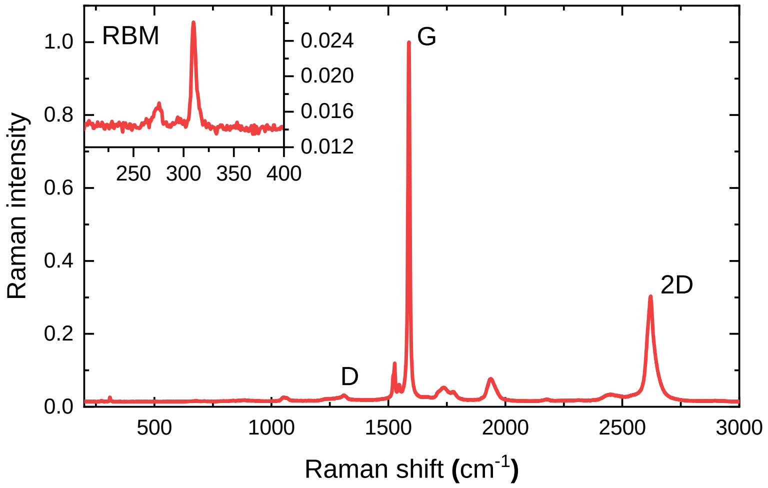

Figure S4. Raman spectrum of the M-(6,5) semiconducting SWCNT sample. The observation of the RBM mode represents the measurements of single-wall carbon nanotubes. The low D band represents high quality pristine structure was obtained. The Raman system integrated a BX41 Olympus upright microscope with a LabRAM HR800 Horiba confocal Raman and high-resolution spectrometer system. The excitation beam from a 532 nm diode-pumped solid-state laser (Excelsior-532-100-CDRH, Spectra-Physics) was first purified by corresponding laser-line filters and then focused via an objective lens (Olympus MPlanFL N 20x/0.45). The backward-scattered radiation was collected via the same objective and passed through corresponding Raman filters to block the reflected laser beam. Then, the radiation was dispersed by an 80-cm spectrometer and recorded by a liquid-nitrogen-cooled CCD (Symphony II, Horiba). The spectral resolution was less than 0.6 cm^{-1}.

Section 2. Optical Stark effects in semiconducting SWCNTs

The optical transitions in semiconducting SWCNTs are dominated by the exciton transition due to formation of strongly bound electron-hole pairs. The interactions between the negatively detuned driving pump photons and exciton states are commonly framed within the Floquet theory, which states that a Hamiltonian periodic in time has quasi-static eigenstates that are evenly spaced in unit of driving pump photon energy[1,2]. In our pump-probe spectroscopy measurements, since we measure the changes of the exciton resonance induced by the negatively detuned driving pump photons ($\Delta E = E_p - E_X$), we therefore focus on the excitonic Floquet states originating from the hybridization between Floquet states $|g,(n+1)\hbar\omega\rangle$ and bare exciton state $|X,n\hbar\omega\rangle$ under driving pump excitations, where $|g\rangle$ and $|X\rangle$ are the ground and exciton states, respectively, and ω is the pump photon frequency ($E_\mathrm{p} = \hbar\omega$). Such interactions can be described by using the standard Jaynes-Cummings model commonly employed to describe two-level atomic system through the following Hamiltonian[2–4]:

$$\hat{H} = \frac{1}{2}\left(E_X - E_g\right)\hat{\sigma}_z + \hbar\omega\hat{a}^+\hat{a} + \frac{1}{2}\hbar\Omega_r(\hat{\sigma}^+\hat{a} + \hat{\sigma}^-\hat{a}^+) \qquad \text{equation S1}$$

where the three terms describe the two-level system, the photon reservoir, and the exciton-photon interactions, respectively. $\hbar\Omega_r$ is the Rabi frequency that quantify the exciton-photon coupling strength [2–4]. E_X and E_g is the energy of $|X\rangle$ and $|g\rangle$, respectively. The Pauli matrices and the ladder operators are given by:

$$\hat{\sigma}_z = |X\rangle\langle X| - |g\rangle\langle g|$$

$$\hat{\sigma}^- = |g\rangle\langle X|$$

$$\hat{\sigma}^+ = |X\rangle\langle g|$$

$$\hat{a}|n\rangle = \sqrt{n}|n-1\rangle$$

$$\hat{a}^+|n\rangle = \sqrt{n+1}|n+1\rangle$$

Within the interaction term, the Hamiltonian couples states $|g,(n+1)\hbar\omega\rangle$ and $|X,n\hbar\omega\rangle$. Using these states as basis, the interaction Hamiltonian matrix can be written as

$$H_{int} = \hbar\omega\left(n+\frac{1}{2}\right)\begin{pmatrix}1 & 0\\ 0 & 1\end{pmatrix} + \frac{1}{2}\begin{pmatrix}E_X - E_g - \hbar\omega & \hbar\Omega_r\sqrt{n+1}\\ \hbar\Omega_r\sqrt{n+1} & -(E_X - E_g - \hbar\omega)\end{pmatrix} \quad \text{equation S2}$$

Direct diagonalization of the Hamiltonian yields the energies of the two new eigenstates:

$$E_{n,\pm} = \hbar\omega\left(n+\frac{1}{2}\right) \pm \frac{\sqrt{(\hbar\Omega_r)^2(n+1)+(E_X-\hbar\omega)^2}}{2} \quad \text{equation S3}$$

As can be seen from equation S3, the excitonic Floquet states emerge through the discrete energy at the interval of $\hbar\omega$ (the frequency of driving pump E_p). When the driving pump (E_p) is on resonant with the exciton transition (E_X), quantum hybridization leads to splitting doublet in the excitonic Floquet band[1–5]. Experimentally, we employed negatively detuned driving pump to manipulate the excitonic Floquet states. In the weak field approximation, where $\sqrt{n}\hbar\Omega_r \ll E_X - E_\mathrm{p}$, equation S3 suggests that the excitonic Floquet state is blueshifted from the bare exciton state (Figure S5a), and can be approximated as:

$$\Delta E_X \approx \frac{1}{2}\frac{n(\hbar\Omega_r)^2}{E_X - E_\mathrm{p}} \propto \frac{I_\mathrm{pump}}{E_X - E_\mathrm{p}} \quad \text{equation S4}$$

The photo-induced Floquet states have been widely observed in several systems using optical pump-probe spectroscopy. This technique enables direct observation of the pump-induced change of optical resonance arising from the optical Stark effects, where the resulting Floquet states energy varies with the driving pump intensity and detuning energy[4–8]. The energy shift gives rise to derivative-like pump-probe features near the exciton resonance[4–7], which is a clear signature of the emergence of excitonic Floquet states, as illustrated in Figure S5.

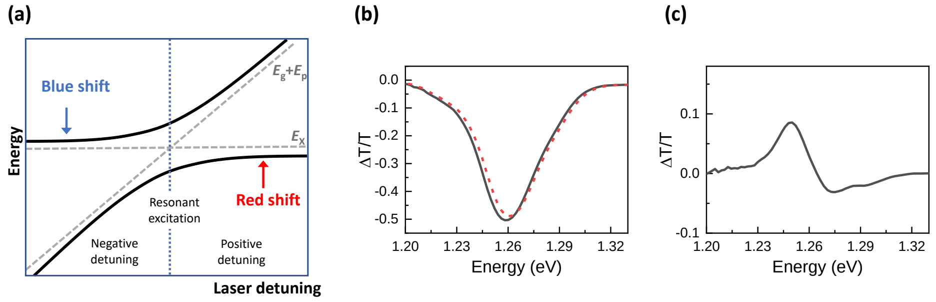

Figure S5. Optical Stark effects and excitonic Floquet states. **(a)** Schematic diagram illustrating the avoided-crossing behavior due to quantum mechanical coupling between the driving pump photons and the excitonic transition. The dashed lines show the energy of the bare exciton state $|X\rangle$ and unperturbed Floquet state $|g, n\hbar\omega\rangle$ as a function of the laser detuning energy ΔE. The blue vertical dotted line shows the resonant excitation ($\Delta E = 0$). The solid lines show the dressed-exciton states from quantum hybridization. In the weak field approximation, where $\sqrt{n}\hbar\Omega_r \ll |\Delta E|$, the optical transition is dominated by the excitonic Floquet state that is blueshifted or redshifted from the bare exciton state (indicated by blue and red vertical arrows, respectively), depending on the sign of ΔE. **(b)** Measured absorption of SWCNT (black) and blueshifted spectrum (dashed red line) resulting from the optical Stark effect. **(c)** The simulated change of transmission spectrum induced by the pump laser.

At $\tau = 0$ ps, the pump-probe signals are dominated by energy blueshift of exciton resonance, which can be estimated by taking a derivative of the absorption spectrum, as shown in Figure S6a. From the fitting, we extract an optical Stark shift of ~2.2 meV. The optical Stark shift scales inversely with the detuning energy and increase with increasing the driving pump intensity, as shown in Figure S6b. The data can be well-described by equation S4. The resonance of the excitonic Floquet state $E_{X'}$ arise from the optical Stark effects is given by $E_{X'} = E_{X_o} + \Delta E_X$. To fit the experimental data, equation S4 can be written as $\Delta E_X = \frac{s*I_{\text{pump}}}{E_X - E_{\text{p}}}$, where s is the fitting constant and I_{pump}, E_X, and E_{p} can be determined experimentally.

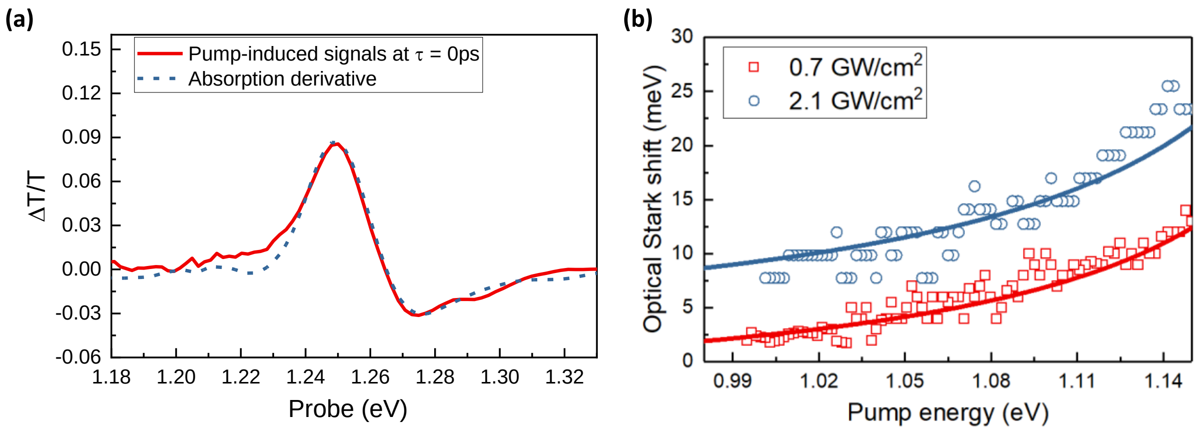

Figure S6. Optical Stark effects in semiconducting SWNTs. **(a)** Pump-probe spectrum at $\tau = 0$ ps (solid lines) at a pump energy of 1.00 eV. The pump intensity is 0.7 GW/cm^2. The observed spectrum matches well with the derivative of linear absorption spectrum of SWNTs (dashed-lines), which corresponds to a blueshift of ~2.2 meV of absorption spectrum. **(b)** The optical Stark shift at various pump excitation energy. The blue circles and red square are data extracted from the pump-probe spectra at excitation intensity of 0.7 GW/cm^2 and 2.1 GW/cm^2, respectively. Solid lines are fitting to the experimental data based on equation S4.

Section 3. Extracting the Raman spectrum from the pump-probe signals

In our pump-probe experiments, the negatively detuned driving pump gives rise to both optical Stark effects and stimulated Raman scattering. To extract the Raman signals, we take a linecut vertically along the probe axis. Besides the noise in the probe spectrum, the background in each linecut is mainly contributed by the signals arising from excitonic optical Stark effects, which varies gradually across the pump energies at each probe wavelength. The noise level increases to around ΔT/T ~ 0.2% when the probe photon energy is lower than 1.29 eV since we detected the optical responses using CCD line camera. Figure S7 shows the pump-induced Raman signals at different scattering frequencies E_{as} at τ = 0 ps. To retrieve the sharp Raman features, we follow the approach in ref. [9] by subtracting the broad background with a polynomial fitting. For G-mode phonon Raman, we approximated the background using a polynomial fitting in the frequency range 1190-2200 cm^{-1}, excluding the phonon region of 1310-1970 cm^{-1}. Similar procedures were applied for 2D mode phonon Raman, where we approximated the background using a polynomial fitting in the frequency range 2200-3000 cm^{-1}, excluding the phonon region of 2450-2850 cm^{-1}.

Figure S7 shows the extracted G-modes Raman spectra at different outgoing Raman scattering resonance E_{as}. In-addition to the G-modes phonon Raman, a weak Raman peak at energy closed to 1360 cm^{-1} can be observed when E_{as} is tuned close to exciton resonance $E_{X'}$, which can be assigned to D-modes phonon Raman in SWCNTs, in-accordance to previous studies.[10,11] This mode shows much weaker Raman intensity than the G-mode phonon Raman, as expected from high quality SWCNTs samples.[10–12] When E_{as} is tuned away from $E_{X'}$ (Figure S7c, and S7d), the D-mode Raman signal becomes indistinguishable in the spectrum, presumably suppressed by the destructive interference between the phonon Raman pathways and exciton transitions. These observations demonstrate that our approach in manipulating the Fano resonance using femtosecond pulses can enhance the detection of extremely weak Raman signals in semiconducting nanostructures.

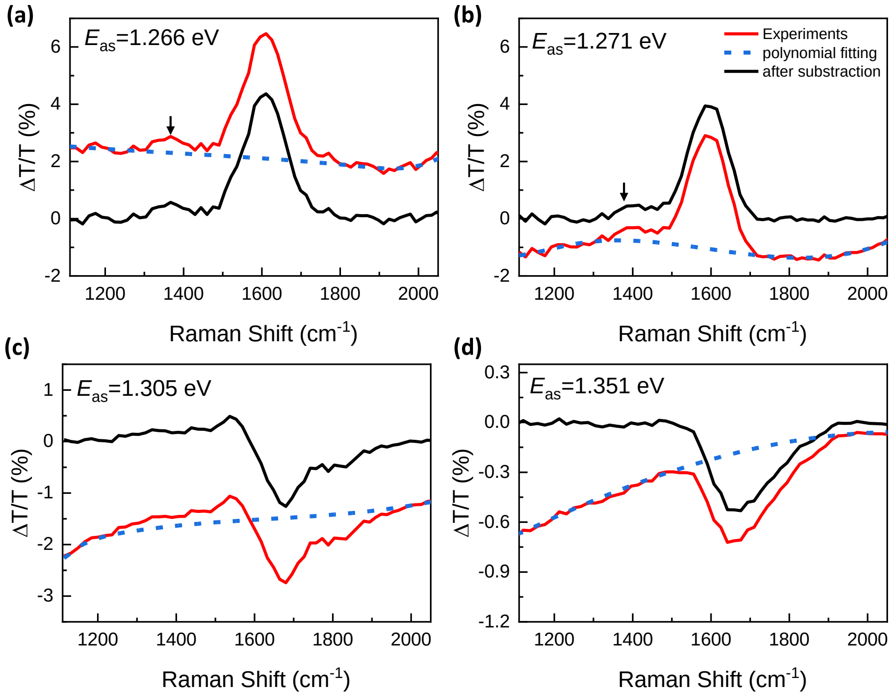

Figure S7. Raman Scattering around G-mode phonons. **a-d.** Pump-induced Raman signals at scattering frequencies E_{as} of (a) 1.266 eV, (b) 1.271 eV, (c) 1.305 eV, and (d) 1.351 eV at $\tau =$ 0 ps (red solid lines). The horizontal axis shows the Raman shift, which corresponds to the difference between pump and probe energy. The broad background was fitted by a polynomial function (blue dashed curves) across the frequency range of 1190-2200 cm^{-1}, excluding the phonon region of 1310-1970cm^{-1}. The Fano lineshapes evolve from antiresonance to dispersive feature and to symmetric Lorentzian, as E_{as} is reduced from 1.351 eV to 1.268 eV. The main peak at energy around 1620 cm^{-1} is attributed to the G-mode phonon Raman. For E_{as} tuned close to exciton resonance, additional peak emerges at energy around 1360 cm^{-1} (indicated by a downward arrow), which can be attributed to D-mode phonon Raman. This mode becomes extremely weak when E_{as} is tuned away from $E_{X'}$.

Section 4. 2D-mode phonon Raman scattering

The 2D-mode phonon Raman scatterings also show prominent Fano lineshape that varies with E_{as}. The Raman scatterings exhibit Fano lineshapes and can be well-described by equation 1 in main text.

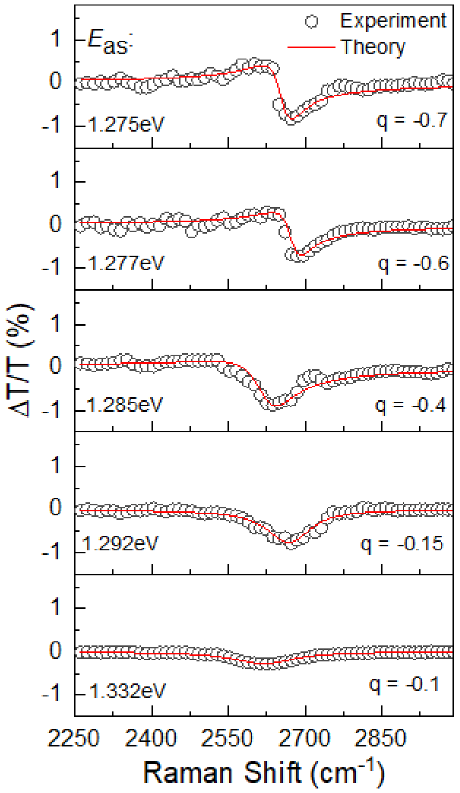

Figure S8. 2D-mode phonon Raman scattering at various scattering frequencies. The driving pump intensity is 0.7 GW/cm^2. The spectra show prominent Fano-lineshapes that can be simulated based on equation 1 in main text (red solid lines). Symbols are experimental data.

Section 5. Driving pump detuning energy- and intensity-dependent Fano interference

The G-mode phonon Raman spectra can be well-described by the Fano lineshapes based on equation 1 in main text. The experimental data were fitted to equation 1, optimized for the G-mode Raman spectrum in the range of 1430-2000 cm^{-1}. The fitting parameters used were I_o, q, $\gamma_{\Omega'}$, and $E_{\Omega'}$.The fitting procedure is similar to the approaches in ref. [9,13].

Figure S9 shows both $E_{\Omega'}$ and $\gamma_{\Omega'}$ extracted from fitting the experimental data shown in Figure 3a to equation 1 in main text. Both $E_{\Omega'}$ and $\gamma_{\Omega'}$ vary with the Raman scattering frequency scattering E_{as} , signifying the hybrid exciton-phonon nature of the Raman intermediate state is strongly modified by the electron-phonon interactions.

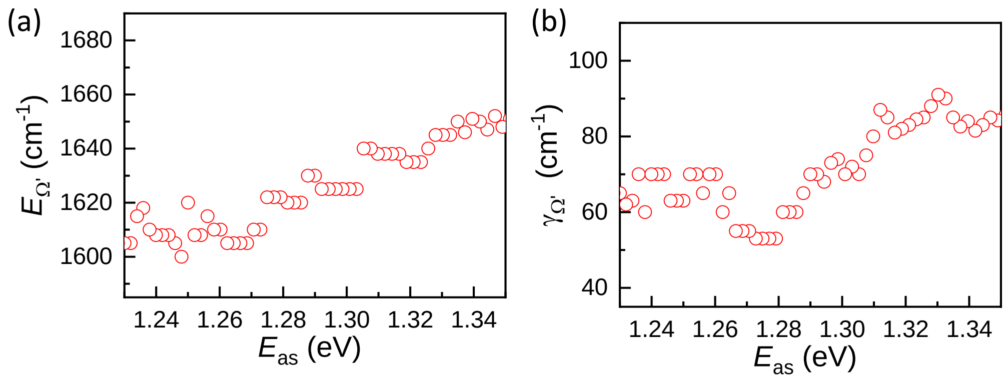

Figure S9. Detuning energy dependence of Fano lineshapes. The extracted center frequency E_{Ω} (a) and linewidth γ (b) of the dressed phonon as a function of scattering frequencies E_{as}. The intensity of the driving pump was kept constant at 0.7 GW/cm^2 across the tuning range.

Figure S10 shows the Fano lineshapes under difference excitation intensities. The lineshapes can be well-described using equation 1 shown in main text. Transition from antiresonance to dispersive and to Lorentzian profile can be clearly observed across different driving intensities. However, there is a subtle difference of such transition – by increasing the driving intensity, transition from asymmetric Fano lineshapes to symmetric Lorentzian profile shifts to higher scattering frequencies (E_{as}), a quantum nonlinear effects that can be quantitatively captured by theoretical models through fitting the experimental data to equation 2 in main text.

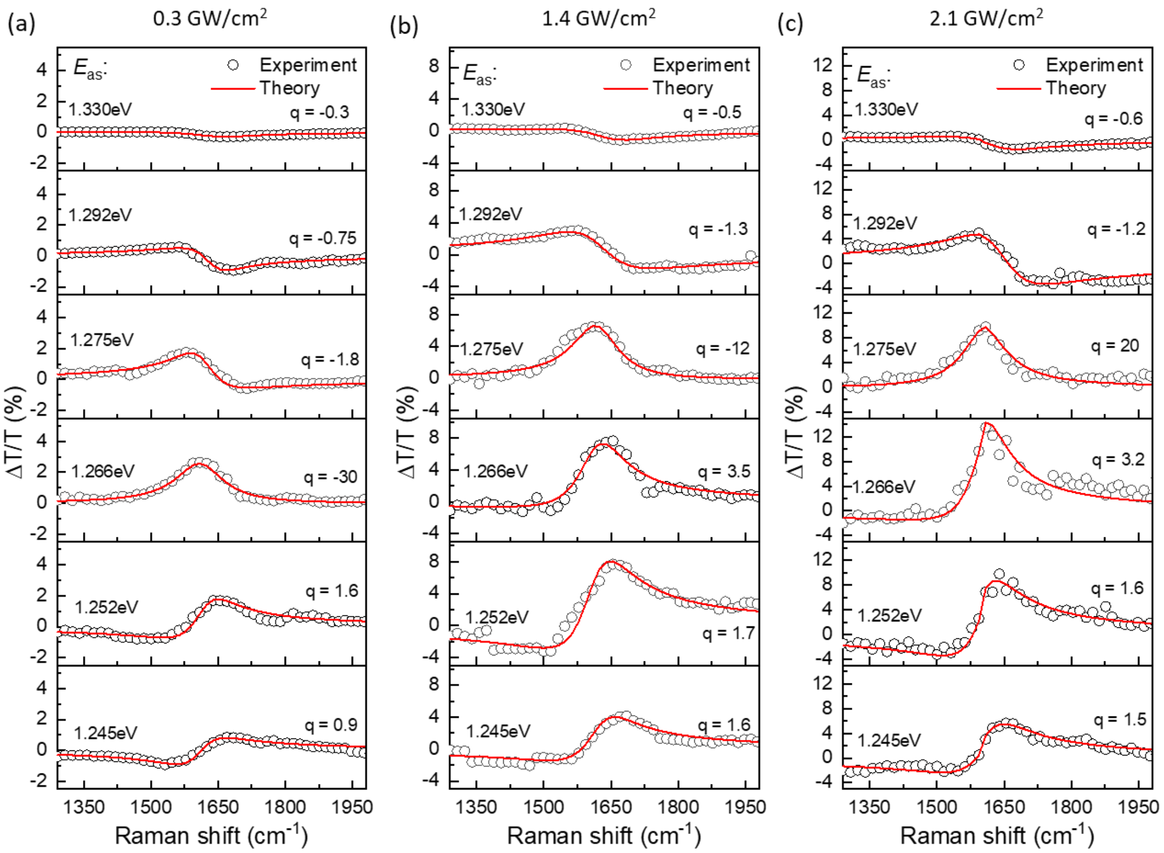

Figure S10. Pump intensity dependence of Raman lineshapes at various scattering frequencies for driving pump intensity kept constant at (a) 0.3 GW/cm^2, (b) 1.4 GW/cm^2, and (c) 2.1 GW/cm^2. The solid red curves are fitting based on equation 1 shown in main text.

Section 6. Fano parameter q

The classical Fano interference emerges when the energy-matched discrete state couples to continuum band in the excited state manifold. From the modified transition lineshape, a phenomenological q factor could be extracted as an indicator of the relative oscillator strength between the continuum states $|\psi_E\rangle$ and pathways affected by the discrete level $|\varphi\rangle$. The Fano parameter q is given by,[14]

$$q = \frac{\langle \Phi | T | g \rangle}{\pi \langle \varphi | H | \psi_E \rangle \langle \psi_E | T | g \rangle} \qquad \text{equation S5}$$

where H and T represents the coupling Hamiltonian and the electron-photon operator respectively, and $|g\rangle$ is the ground state. Here, $|\Phi\rangle$ represents $|\varphi\rangle$ modified by an extra term via coupling with the continuum states:[14]

$$|\Phi\rangle = |\varphi\rangle + \sum_u \frac{\langle \psi_u | H | \varphi \rangle |\psi_u\rangle}{E - E_u} \qquad \text{equation S6}$$

$\langle \Phi | T | g \rangle$ in equation S5 therefore describes the pathways involving the transition to the discrete states dressed by continuum band. E_u denotes the continuum states with energy matching that of $|\Phi\rangle$, respectively.

In our case, the discrete and continuum states referred to the phonon states $|\varphi\rangle \equiv |\Omega\rangle$ and excitonic Floquet resonance $|\psi_E\rangle \equiv |X'\rangle$, respectively. Unlike in classical Fano system, the discrete phonon state in our system is energetically remote from the exciton band. Furthermore, the phonon state is a Raman active mode, where direct optical transition is forbidden. In SRS, interactions between the phonon and excitonic continuum are predominantly mediated by the three field-matter interactions (as depicted in Figure 1c in main text), where the phonon Raman intermediate states $|n\rangle = |g, \Omega, E_{\mathrm{pr}} - E_{\mathrm{p}} + E_{\mathrm{p}}\rangle$ can be tuned to resonantly couple to the excitonic Floquet band. In this case, the phonon-exciton interactions can be described by the

matrix element $\langle X'_u|H_{\mathrm{X-ph}}|\mathrm{n}\rangle$, where $H_{\mathrm{X-ph}}$ is the exciton-phonon coupling Hamiltonian and $|X'_u\rangle$ is the excitonic Floquet state with energy matching that of $|n\rangle$. Equation S6 can then be written as:

$$|\Phi\rangle = |\Omega\rangle + \sum_u \frac{\langle X'_u|H_{\mathrm{X-ph}}|\mathbf{n}\rangle|X'_u\rangle}{E - E_u} \qquad \text{equation S7}$$

Inserting equation S7 into equation S5, the Fano parameter q can be written as:

$$q = \frac{\sum_u \frac{\langle \mathrm{n}|H_{\mathrm{X-ph}}|X'_u\rangle\langle X'_u|T|g\rangle}{E - E_u}}{\pi D(E)\langle \mathrm{n}|H_{\mathrm{X-ph}}|X'\rangle\langle X'|T|g\rangle} \qquad \text{equation S8}$$

where the term $\langle \Omega|T|g\rangle$ diminishes because the phonon modes interact with the electronic states exclusively through $H_{\mathrm{X-ph}}$. $\langle \mathrm{n}|H_{\mathrm{X-ph}}|X'\rangle$ represents the exciton-phonon coupling V_{K}. The joint electron-hole pair density of states $D(E)$ is added since the continuum wavefunctions in our case are normalized to unity.[15] In equation S8, the numerator described the matrix elements linked to SRS within the context of second order perturbation theory, where $\sum_u \frac{\langle \mathrm{n}|H_{\mathrm{X-ph}}|X'_u\rangle\langle X'_u|T|g\rangle}{E-E_u}$ is the summation over the second-order perturbation contributions to the overall transition amplitude.[16–19] When the energy of the outgoing Raman scattered photon E_{as} is tuned close to the excitonic Floquet resonance, the matrix elements weighted by the joint electron-hole pair density of states can be approximated as M_Ω[16–19]:

$$M_\Omega \sim \sum_u D(E_u) \frac{\langle \mathrm{n}|H_{\mathrm{X-ph}}|X'_u\rangle\langle X'_u|T|g\rangle}{E - E_u}$$

$$= \frac{B_{\mathrm{k}}}{\left((E_{X'} - (E_\Omega + E_{\mathrm{p}}) - i\gamma_\Omega\right)\left(E_{X'} - E_{\mathrm{pr}} - i\gamma_X\right)} \qquad \text{equation S9}$$

where γ_{Ω} and γ_{X} are the linewidths of bare phonon and exciton, respectively. $D(E_u)$ is the joint electron-hole pair density of states at the $|n\rangle$ energy. The parameter B_{k} is related to the exciton-phonon coupling, exciton-photon coupling and density of states.

On the other hand, $\langle X'|T|g\rangle$ in equation S8 describe the interband transition. Close to the exciton resonance, the matrix element for interband transition can be expressed as:[15,20]

$$M_X = \frac{V_{\mathrm{X}}}{E_{X'} - E_{\mathrm{pr}} - i\gamma_X} \qquad \text{equation S10}$$

where V_{X} is the exciton-photon coupling. As a result, the Fano factor q can be reduced to the form of equation (2) in the main text as $q = \frac{1}{\pi D(E) V_{\mathrm{k}}} \times \frac{M_{\Omega}}{M_X}$.

References

[1] J. H. Shirley, *Phys. Rev.* **1965**, *138*, B979.
[2] S. H. Autler, C. H. Townes, *Phys. Rev.* **1955**, *100*, 703.
[3] E. T. Jaynes, F. W. Cummings, *Proceedings of the IEEE* **1963**, *51*, 89.
[4] C.-K. Yong, M. I. B. Utama, C. S. Ong, T. Cao, E. C. Regan, J. Horng, Y. Shen, H. Cai, K. Watanabe, T. Taniguchi, S. Tongay, H. Deng, A. Zettl, S. G. Louie, F. Wang, *Nature Materials* **2019**, *18*, 1065.
[5] C.-K. Yong, J. Horng, Y. Shen, hui Cai, A. Wang, C.-S. Yang, C.-K. Lin, S. Zhao, K. Watanabe, T. Taniguchi, S. Tongay, F. Wang, *Nat. Phys.* **2018**, *14*, 1092.
[6] C. Sieh, T. Meier, F. Jahnke, A. Knorr, S. W. Koch, P. Brick, M. Hübner, C. Ell, J. Prineas, G. Khitrova, H. M. Gibbs, *Phys. Rev. Lett.* **1999**, *82*, 3112.
[7] D. Fröhlich, A. Nöthe, K. Reimann, *Phys. Rev. Lett.* **1985**, *55*, 1335.
[8] A. Mysyrowicz, D. Hulin, A. Antonetti, A. Migus, W. T. Masselink, H. Morkoç, *Phys. Rev. Lett.* **1986**, *56*, 2748.
[9] T.-T. Tang, Y. Zhang, C.-H. Park, B. Geng, C. Girit, Z. Hao, M. C. Martin, A. Zettl, M. F. Crommie, S. G. Louie, Y. R. Shen, F. Wang, *Nature Nanotechnology* **2010**, *5*, 32.
[10] A. Jorio, C. Fantini, M. S. S. Dantas, M. A. Pimenta, A. G. Souza Filho, Ge. G. Samsonidze, V. W. Brar, G. Dresselhaus, M. S. Dresselhaus, A. K. Swan, M. S. Ünlü, B. B. Goldberg, R. Saito, *Phys. Rev. B* **2002**, *66*, 115411.
[11] A. G. Souza Filho, A. Jorio, G. Dresselhaus, M. S. Dresselhaus, R. Saito, A. K. Swan, M. S. Ünlü, B. B. Goldberg, J. H. Hafner, C. M. Lieber, M. A. Pimenta, *Phys. Rev. B* **2001**, *65*, 035404.
[12] F. Wang, W. Liu, Y. Wu, M. Y. Sfeir, L. Huang, J. Hone, S. O’Brien, L. E. Brus, T. F. Heinz, Y. R. Shen, *Phys. Rev. Lett.* **2007**, *98*, 047402.
[13] F. Lapointe, É. Gaufrès, I. Tremblay, N. Y.-W. Tang, R. Martel, P. Desjardins, *Phys. Rev. Lett.* **2012**, *109*, 097402.
[14] U. Fano, *Phys. Rev.* **1961**, *124*, 1866.
[15] M. V. Klein, in *Light Scattering in Solids* (Ed: M. Cardona), Springer, Berlin, Heidelberg, **1975**, pp. 147–204.
[16] A. Jorio, A. G. Souza Filho, G. Dresselhaus, M. S. Dresselhaus, R. Saito, J. H. Hafner, C. M. Lieber, F. M. Matinaga, M. S. S. Dantas, M. A. Pimenta, *Phys. Rev. B* **2001**, *63*, 245416.
[17] P. Y. Yu, M. Cardona, in *Fundamentals of Semiconductors: Physics and Materials Properties* (Eds: P.Y. Yu, M. Cardona), Springer Berlin Heidelberg, Berlin, Heidelberg, **2010**, pp. 345–426.
[18] Y. R. Shen, N. Bloembergen, *Phys. Rev.* **1965**, *137*, A1787.
[19] C.-S. Wang, *Phys. Rev.* **1969**, *182*, 482.
[20] H. Kalt, C. F. Klingshirn, in *Semiconductor Optics 1: Linear Optical Properties of Semiconductors* (Eds: H. Kalt, C.F. Klingshirn), Springer International Publishing, Cham, **2019**, pp. 329–385.
